# Plasmid Complement of *Lactococcus lactis* NCDO712 Reveals a Novel Pilus Gene Cluster

**DOI:** 10.1371/journal.pone.0167970

**Published:** 2016-12-12

**Authors:** Mariya Tarazanova, Marke Beerthuyzen, Roland Siezen, Marcela M. Fernandez-Gutierrez, Anne de Jong, Sjoerd van der Meulen, Jan Kok, Herwig Bachmann

**Affiliations:** 1 NIZO food research B.V., Ede, The Netherlands; 2 TI Food and Nutrition, Wageningen, The Netherlands; 3 Molecular Genetics, University of Groningen, Groningen, The Netherlands; 4 Centre for Molecular and Biomolecular Informatics, Radboud UMC, Nijmegen, The Netherlands; 5 Microbial Bioinformatics, Ede, The Netherlands; 6 Host-Microbe Interactomics Group, Animal Sciences, Wageningen University, Wageningen, The Netherlands; Hosei Daigaku, JAPAN

## Abstract

*Lactococcus lactis* MG1363 is an important gram-positive model organism. It is a plasmid-free and phage-cured derivative of strain NCDO712. Plasmid-cured strains facilitate studies on molecular biological aspects, but many properties which make *L*. *lactis* an important organism in the dairy industry are plasmid encoded. We sequenced the total DNA of strain NCDO712 and, contrary to earlier reports, revealed that the strain carries 6 rather than 5 plasmids. A new 50-kb plasmid, designated pNZ712, encodes functional nisin immunity (*nisCIP)* and copper resistance (*lcoRSABC)*. The copper resistance could be used as a marker for the conjugation of pNZ712 to *L*. *lactis* MG1614. A genome comparison with the plasmid cured daughter strain MG1363 showed that the number of single nucleotide polymorphisms that accumulated in the laboratory since the strains diverted more than 30 years ago is limited to 11 of which only 5 lead to amino acid changes. The 16-kb plasmid pSH74 was found to contain a novel 8-kb pilus gene cluster *spaCB-spaA-srtC1-srtC2*, which is predicted to encode a pilin tip protein SpaC, a pilus basal subunit SpaB, and a pilus backbone protein SpaA. The sortases SrtC1/SrtC2 are most likely involved in pilus polymerization while the chromosomally encoded SrtA could act to anchor the pilus to peptidoglycan in the cell wall. Overexpression of the pilus gene cluster from a multi-copy plasmid in *L*. *lactis* MG1363 resulted in cell chaining, aggregation, rapid sedimentation and increased conjugation efficiency of the cells. Electron microscopy showed that the over-expression of the pilus gene cluster leads to appendices on the cell surfaces. A deletion of the gene encoding the putative basal protein *spaB*, by truncating *spaCB*, led to more pilus-like structures on the cell surface, but cell aggregation and cell chaining were no longer observed. This is consistent with the prediction that *spaB* is involved in the anchoring of the pili to the cell.

## Introduction

*Lactococcus lactis* is a gram-positive, non-pathogenic, non-spore forming lactic acid bacterium (LAB) that is often isolated from plant material or a dairy environment [1,2]. It is widely used in the dairy industry as a starter culture for the production of cheese, buttermilk and quark. Strains of *L*. *lactis* typically contain one to eight different plasmids [3,4] ranging from 1 kb to more than 100 kb in size [5,6]. Examples are *L*. *lactis* SK11, a phage-resistant dairy strain used in cheese making that carries 5 plasmids [7], and *L*. *lactis* IL594, the 7-plasmid-containing parent of the plasmid-free laboratory strain IL1403 [8]. The plasmids often specify traits of industrial importance such as the ability to grow on lactose, to utilize milk protein or stress resistance [9,10]. Other important plasmid-encoded functions include bacteriocin production [11,12] and resistance [13,14], metal ion resistance [15], antibiotic resistance [16,17] and bacteriophage resistance [18,19]. A recent publication described a CRISPR-Cas system that was encoded by a lactococcal plasmid, although it was concluded not to be functional [20]. In addition, several genes related to lactococcal surface properties are carried on plasmids [21–23], such as *aggL*, a gene responsible for cell auto-aggregation, or genes responsible for adhesion to mucus [24].

One of the most intensively studied *L*. *lactis* strains is MG1363, a plasmid-cured derivative of strain NCDO712 [25,26]. NCDO712 was originally isolated from a dairy starter culture and was described to harbor 5 plasmids with molecular sizes of approximately 33, 9, 5.2, 2.5, and 1.8 MDa [26]. During plasmid curing of strain NCDO712, derivatives carrying individual plasmids were obtained, allowing a targeted analysis of plasmid-encoded functions [26]. Important biotechnological properties of the strain, namely lactose utilization and proteolysis, were linked to the 33 MDa (55 kb) plasmid pLP712 [26,27].

This lactose/protease plasmid pLP712 [27] can be transferred to other lactococcal strains by conjugation [28]. Conjugation can occur through co-integrate formation between pLP712 and a genome-encoded sex factor (SF). The co-integrate is roughly double the size of pLP712 since the SF is 59.5 kb [29]. After conjugation approximately half of the transconjugants displayed an aggregating phenotype and transferred the lactose/protease plasmid with high-frequency [28,30–33]. The aggregating phenotype is only seen in transconjugants carrying the pLP712-SF co-integrate [29,34] and was linked to the *cluA* gene on the SF [34,35]. This gene encodes a surface protein involved in cell-to-cell contact and cell aggregation [34,35].

Some *L*. *lactis* strains also express proteinaceous surface appendages called pili [21,22,24]. Pili are known to have different functions in bacteria, including adhesion to surfaces (type I pili) or motility (type IV pili) [36–39]. Pilus biosynthesis genes can be encoded by the chromosomal DNA [22] or by plasmids [21] and they are described to be involved in cell aggregation [40], bacterial adherence to host cells [41–43] and attachment to environmental substrates/surfaces [44].

Two plasmids of *L*. *lactis* NCDO712 have been sequenced. Plasmid pLP712 (55395 bp) harbors the genes for lactose import and catabolism, the extracellular protease PrtP, and genes encoding extracellular proteins, transposases, and hypothetical proteins [27]. Plasmid pSH71, the smallest one (2062 bp) [45], is highly similar to pWV01 [45,46] and both of them are the basis of an array of broad host-range cloning and gene expression vectors [45,47–51].

Here we sequenced all plasmids of *L*. *lactis* NCDO712 and found that contrary to earlier reports, it contains not 5, but 6 plasmids [26]. The additional plasmid encodes functional nisin immunity and copper resistance genes. Additionally, we identified a novel pilus gene cluster on plasmid pSH74, which we showed to be functional by over-expression and phenotypic analyses.

## Materials and Methods

### Bacterial strains, growth conditions and medium

*L*. *lactis* subsp. *cremoris* NCDO712 [26] and its derivatives (Table 1) were grown at 30°C in M17 (Oxoid, Thermo Scientific, Hampshire, UK). The lactose-positive *L*. *lactis* strains NDO712 and its derivatives SH4109 and MG1299 were grown in M17 containing 1% lactose (LM17), all other strains were grown in M17 supplemented with 1% glucose (GM17). When required, erythromycin (Ery; 10 μg/ml), chloramphenicol (Cm; 5 μg/ml), rifampicin (Rif; 50 μg/ml), or streptomycin (Str; 100 μg/ml) was added.

**Table 1:** Strains and plasmids used in this study.

Strain or plasmid	Characteristics^#^	Reference
***L*. *lactis* strains**		
NCDO712	*L*. *lactis* dairy isolate (harboring pLP712, pSH71, pSH72, pSH73, pSH74, pNZ712)	[26]This study
MG1363	Plasmid-cured derivative of *L*. *lactis* NCDO712	[26]
SH4109	Prophage-cured derivative of *L*. *lactis* NCDO712 containing all 6 plasmids found in this study	[26]
MG1388	A phage T712 lysogen derived from *L*. *lactis* MG1363	[26]
MG1362	Derivative of *L*. *lactis* NCDO712 (harbors pSH72)	[26]
MG1063	Derivative of *L*. *lactis* NCDO712 (harbors pSH73 and pSH72)	[26]
MG1261	Derivative of *L*. *lactis* NCDO712 (harbors pSH73)	[26]
MG1365	Derivative of *L*. *lactis* NCDO712 (harbors pSH71)	[26]
MG1299	Derivative of *L*. *lactis* NCDO712 (harbors pLP712)	[26]
NZ9700	Nis^R^; Derivative of *L*. *lactis* MG1363; *pepN*::*nisRK*	[48]
MG1614	Str^R^ and Rif^R^ derivative of *L*. *lactis* MG1363	[26]
IL1403	Plasmid-free derivative of *L*. *lactis* IL594	[1]
**Plasmids**		
pIL253	Ery^R^; 4.9kb; Low copy-number derivative of pAMβ1	[52]
pIL25*3pil*	Ery^R^; 13.1kb; pIL253 harboring pSH74 pilus gene cluster *spaCB-spaA-srtC1-srtC2* with 300 bp upstream region	This study
pIL253*pilΔ1*	Ery^R^; 11.6 kb; pIL253 harboring *spaCB-spaA-srtC1-srtC2* with 1,5-kb internal deletion in *spaCB*	This study

^#^ Str^R^—streptomycin resistant, Rif^R^—rifampicin resistant, Ery^R^—erythromycin resistant, Nis^R^—nisin resistant

### DNA sequencing and sequence assembly

Total DNA of *L*. *lactis* NCDO712 was isolated using phenol-chloroform extraction as previously described [53] with the following modifications. Exponentially growing cells were harvested by centrifugation (10 min at 6240 g) after which the cell pellet was re-suspended in THMS buffer (30 mM Tris-HCL (pH 8), 3 mM magnesium chloride, 25% sucrose) containing lysozyme (2 mg/ml) and 50 μg/ml RNase and incubated for 1 h at 37°C. Subsequently, the cells were treated with SDS (at 1% final concentration) for 20 min at 65°C. Next, proteinase K (0.3 mg/ml) was added and incubation was continued for 10 min at 37°C. Total DNA was extracted from the lysate using several extractions with phenol/chloroform after which the DNA was precipitated with isopropanol, was dissolved in sterile water and stored at 4°C.

The purified total DNA was sheared to fragments of approximately 500 bp using the Covaris ultrasone device (KBioscience, LGC, Köln, Germany). The paired-end NEB NExtGen library preparation kit (New England Biolabs, Inc., MA, US) was used according to the manufacturer’s instructions to prepare NGS libraries. The libraries were sequenced on an Illumina HiSeq2000 (Illumina, Inc., San Diego, CA, USA), providing paired-end sequences of 101 bases. Velvet [54,55] was used in combination with VelvetOptimiser (http://bioinformatics.net.au/software.velvetoptimiser.shtml) to perform *de novo* paired-end assembly of the genome. All contigs that did not map to the published nucleotide sequence of the *L*. *lactis* MG1363 genome (Accession: NC_009004.1) were assumed to be plasmid fragments; these were first scaffolded by mapping onto known *L*. *lactis* plasmids in the NCBI database. Further scaffolding was supported by PacBio sequencing (BaseClear, Leiden, The Netherlands) on a 5-kb library of NCDO712 total DNA. Remaining gaps in the plasmid sequences were closed with dedicated PCR reactions followed by amplicon sequencing (BaseClear, Leiden, The Netherlands).

Initial automatic annotation of the plasmids was performed using the RAST annotation server [56]. Manual curation of plasmid-encoded features was performed with Artemis [57,58], followed by family, domain, motif and context analyses of encoded proteins using BlastP (NCBI) and Interpro (http://www.ebi.ac.uk/interpro/). IS elements and transposase genes were identified using IS Finder (https://www-is.biotoul.fr//). The DNA sequences of the assembled plasmids were used for a BlastN search (http://blast.ncbi.nlm.nih.gov/Blast.cgi) in the NCBI plasmid database containing complete plasmids. Determination of single nucleotide polymorphisms (SNPs) in the nucleotide sequence of the chromosome of strain NCDO712 relative to that of its derivative strain MG1363 was performed using the Breseq software package [59] and the GenBank file: NC_009004 in combination with corresponding next-generation sequencing data: SRA064225 as templates.

For non-synonymous SNPs the software SIFT [60] and the UniProt-TrEMBL database (http://www.uniprot.org/) were used to predict whether an amino acid substitution would affect protein function.

The nucleotide sequences of the pSH74 and pNZ712 plasmids were deposited in the NCBI GenBank database with accession numbers KX138410 and KX138409, respectively.

### Determination of nisin and copper resistance

Overnight cultures of *L*. *lactis* were diluted in fresh GM17 medium to a final optical density at 600 nm (OD_600_) of 0.03. To measure nisin resistance, nisin from *L*. *lactis* (N6764-5G, Sigma-Aldrich, Steinheim, Germany) was added to the medium to different end concentrations (0–20 ng/ml). *L*. *lactis* NZ9700 (Table 1) was used as a control. The strains were grown in 10 ml sterile tubes for 7 h at 30°C. The OD_600_ was measured after 4 h and after 7 h using a UV/Visible Ultrospec 2000 spectrophotometer (Pharmacia Biotech, Cambridge, England).

To measure copper resistance, CuSO_4_ (0–4.8 mM end concentrations) was added to the growth medium. A 96-well microplate with the samples was incubated for 21 h at 30°C. The OD_600_ was measured every 15 min with a SpectraMax spectrophotometer (Molecular Devices, Wokingham, UK). The minimum inihibitory concentration (MIC) of copper was determined as the concentration of CuSO_4_ (g/L) that did not result in visible growth after 15 hours of incubation.

### Pilin overexpression in *L*. *lactis*

The *spaCBA-srtC1-srtC2* locus (designated as *pil* locus) including its 300-bp upstream region was amplified with KOD Hot Start Polymerase (Merck Millipore, Madison, Wisconsin, USA) using the pilinPstI forward and the pilinXhoI reverse primers (S1 Table). The purified PCR product was digested with PstI and XhoI and ligated to similarly digested pIL253 using T4 DNA Ligase (Invitrogen, Breda, The Netherlands). The ligation mixture was used to transform [61] electro-competent [62] MG1363 cells. Transformants harbouring the anticipated pIL253*pil* plasmid (Table 1) were identified performing colony PCR with primers pSH74_FW and pSH74_RV (S1 Table).

An internal deletion of 1451 bp in the *spaCB* gene was constructed by digestion of pIL253*pil* with AatII followed by re-ligation and introduction of the plasmid in *L*. *lactis* MG1363. The resulting plasmid was designated pIL253*pilΔ1*. Transformation of plasmids into other strains was performed with the protocol described above.

### Cell aggregation

*L*. *lactis* cells from a 10-ml overnight culture were washed twice with 10 ml sterile 10 mM phosphate buffer (pH 6.8) and re-suspended in the same buffer. Cell sedimentation was observed visually and cell chaining was examined using bright field microscopy.

### Scanning Electron Microscopy (SEM)

Bacterial cells were cultured for 1 day on GM17 agar plates. From plates with 50–100 colonies, small pieces of agar gel carrying less than 5 colonies were cut out and placed in a microscope sample holder. All further steps of cell fixation, washing, dehydration, staining, freeze-drying, electron microscopy, and image analysis were performed according to [63]. A FEI Magellan 400 FESEM electron microscope (Wageningen Electron Microscopy Centre, The Netherlands) was used for imaging.

### Conjugation

Conjugation experiment were performed as described previously [64,65] with *L*. *lactis* MG1614 as the recipient strain. Transconjugants were selected on milk agar plates containing 0,004% bromocresol purple (Merck, Darmstadt, Germany), streptomycin (100 μg/ml) and rifampicin (50 μg/ml) with the donor strains NCDO712(pIL253*pil*) or MG1299(pIL253*pil*). LM17 plates supplemented with streptomycin (100 μg/ml), rifampicin (50 μg/ml) and 1.2 mM CuSO_4_ were used for the conjugative transfer of pNZ712, carrying the copper resistance genes, from *L*. *lactis* NCDO712 to *L*. *lactis* MG1614.

## Results and Discussion

### Chromosomal differences between *L*. *lactis* strains NCDO712 and MG1363

Sequencing of the total DNA of NCDO712 allowed detecting 11 Single Nucleotide Polymorphisms (SNPs) between the chromosomes of *L*. *lactis* NCDO712 and its plasmid-cured derivative MG1363 [66]. The latter was isolated in 1983 following multiple rounds of chemical- and protoplast-induced plasmid curing [26]. Among the 11 SNPs in the chromosome of *L*. *lactis* NCDO712, three are synonymous and three are in intergenic regions, while the other five lead to amino acid changes in proteins (S2 Table). The nucleotide sequencing data also suggests the occurrence of genome re-arrangements mainly due to mobile genetic elements, but their verification was outside the scope of this study.

Only one of the three SNPs in the intergenic regions is predicted to be in a promoter region, that of the *mtlA* gene encoding a putative mannitol-specific PTS system EIIBC component. Differential RNA sequencing has pinpointed the transcription start site (TSS) of *mtlA* at position 26465 [67]. The mutation at the position 26455 suggests that the -10 box is altered from an optimal TATAAT into TACAAT. Furthermore, three of the protein sequence-affecting SNPs in the genes encoding a hypothetical protein and two transposases were predicted not to affect protein functions. These predictions were made using SIFT, an algorithm that analyzes the effect of mutations based on the degree of conservation of amino acid residues [60,68]. Mutation in the *gapB* and *tsf* genes encoding the glyceraldehyde 3-phosphate dehydrogenase and elongation factor TS, respectively, were predicted to affect protein function. Whether these mutations are caused by genetic drift or whether they might confer a fitness advantage in a laboratory environment is unclear. However, the data indicate that the number of SNPs that accumulated since the isolation of MG1363 more than 30 years ago is limited.

MG1363 is also described to have lost a ~40 kb DNA fragment during its generation from NCDO712, which rendered MG1363 insensitive to UV induced lysis [69]. Based on this information we expected to find this additional ~40 kb fragment in the genome of NCDO712 when comparing the sequence to MG1363. Because we were not able to identify such a fragment we tried to induce lysis in NCDO712 by treatment with either mitomycin C or exposure to UV radiation. With neither approach we were able to induce lysis in NCDO712, suggesting that the variant we were working with did spontaneously loose this fragment. A NCDO712 variant without this 40 kb fragment is phenotypically the same as strain SH4109. However due to potential genome rearrangements in our NCDO712 isolate and the known high genome plasticity in this strain and its derivatives, it cannot be concluded that our isolate is identical to SH4109.

### *L*. *lactis* NCDO712 harbors six plasmids

Assembly of all nucleotide sequence reads that did not map onto the chromosome of *L*. *lactis* MG1363 revealed that *L*. *lactis* NCDO712 carries a total of 6 rather than the previously described 5 plasmids [26]. Using *L*. *lactis* NCDO712 derivatives harboring single plasmid species [26], we linked the plasmids identified here to the earlier described plasmids of this strain (Table 2). The published plasmid sizes did not fully correspond to the respective sizes determined here. This could be caused by the limitations of size estimation based on agarose gel electrophoresis used previously [26]. However, we cannot exclude that plasmid rearrangements have occurred during strain propagation over the years that could account for (part of) the differences, although we did not observe such genetic changes in the 55.4-kb pLP712 plasmid (see below) [27]. The additional plasmid identified in this study, designated pNZ712, has a size similar to that of pLP712, which may explain why it escaped detection in 1983 [26].

**Table 2:** Comparison of NCDO712 plasmids with earlier studies.

Plasmids described in [26]		Plasmids analyzed in this study			
Plasmid	Size^1^	Plasmid	Size	Plasmid copy number/mean coverage^2^	Replication mode
pLP712	33 MDa, ~50 kb	pLP712	55 395 bp	2 (423)	Theta
pSH71^3^	1,8 MDa, ~3 kb	pSH71	2 062 bp	3 (673)	RCR
pSH72^4^	2,5 MDa, ~4 kb	pSH72	3 597 bp	4 (921)	Theta
pSH73^5^	5,2 MDa, ~8 kb	pSH73	8 663 bp	3 (674)	Theta
pSH74	9 MDa, ~14 kb	pSH74	15 518 bp	3 (697)	Theta
_	_	pNZ712	49 832 bp	2 (471)	Theta

^1^ Plasmid size in original publication is given in MDa (1MDa ds-DNA = 1.52 kb (https://tools.thermofisher.com).

^2^ Estimated on the bases sequence coverage in comparison to that of the chromosomal DNA. Coverage number is based on the analysis of 6 million sequence reads. The mean chromosomal DNA coverage in the same analysis was 198.

^3^ pSH71 in this study differs at 6 positions from the publically available sequence of pSH71 (NCBI accession number A09339; de Vos W.M., 1987): T712-deleted, T713-deleted, A731-deleted, G803A, T1234-inserted, C1414-deleted (the nucleotide before the position number indicates the sequence in the plasmid sequenced here, the description after the position number indicates the sequence in accession number A09339).

^4^ pSH72 differs by 3 bp from pND324 [70] (NCBI reference sequence: NC_008436.1): T1295G, G1384A and C3349-deleted. pSH72 is 99% identical to pND324

^5^ pSH73 is 100% identical to pAG6 (NCBI acession number: AB198069, GI: 70067197)

The copy number of the individual plasmids varied between 2 and 4, based on mean coverage number of chromosomal DNA and plasmid coverage (Table 2). The plasmid replication mode was determined using previously described criteria (2, 53). Rolling circle replication (RCR) was identified on the basis of the presence of Rep-family protein encoding genes and a double-stranded origin (*dso*) of replication, while a replication initiator protein-encoding *repB* gene and an origin of replication (*ori*) are indicative of theta-type plasmid replication [9]. These analyses indicate that pSH71 replicates through a rolling circle mechanism [9,45], while the other 5 plasmids replicate using a theta-type mechanism [9].

Plasmids pLP712 and pSH71 have been sequenced and described earlier [27,45]. The nucleotide sequence of the pLP712 is identical to the one determined here, except for a single nucleotide difference, whereas the pSH71 sequence differs by 6 nucleotides (Table 2).

**Fig 1:**
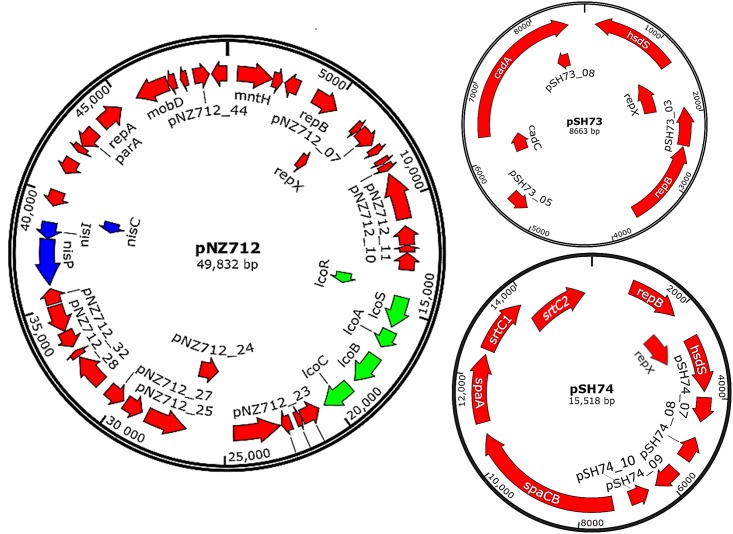
Plasmid maps of pSH73, pSH74, and pNZ712. Plasmid pNZ712 includes genes encoding functional nisin immunity (*nisCIP*) and copper resistance (*lcoRSABC*). The 16-kb plasmid pSH74 contains a novel 8-kb pilus gene cluster *spaCB-spaA-srtC1-srtC2*. Plasmid pSH73 harbors *repX*, *repB* and *cadCA* genes. The latter genes were annotated as a cadmium resistance regulatory protein and a cadmium efflux ATPase.

To investigate the relationships of plasmids pSH72, pSH73, pNZ712, and pSH74 with other known plasmids we compared their nucleotide sequences with 1955 plasmid sequences in the NCBI database (database consulted on February 1, 2015). pSH72 (3597 bp) has the highest copy number, approximately 4 copies per cell, and only appears to encode the replication genes *repB*, *repX*, and *repC*. Except for 3 nucleotide differences pSH72 is identical to plasmid pND324 isolated from *L*. *lactis* subsp. *lactis* LL57-1 [70] (Table 2). The biological function of this plasmid is unclear. pSH73 is identical to pAG6, a plasmid isolated from *Lactococcus lactis* ssp. *cremoris* 712, which is most likely the same strain as NCDO712 or a derivative of it [29]. The only SNP (A → G, pAG6 → pSH73) is at nucleotide position 1143 of the *hsdS* gene encoding a type-I restriction/modification system specificity subunit. This SNP is silent and does not change the amino acid sequence. Plasmid pSH73 harbors, next to the replication genes *repX* and *repB*, also *cadCA* genes that are predicted to encode a cadmium resistance regulatory protein and a cadmium efflux ATPase (Fig 1 and S3 Table).

The two other plasmids pSH74 and pNZ712 have partial similarity (93–99% nucleotide identity) to known *L*. *lactis* plasmids. These high identities were observed in genes encoding several functions such as replication, transposase, resolvase, and in copper resistance-associated genes and in the *nisCIP* genes (Fig 1, S1 Fig and S4 Table). Detailed sequence analysis of pSH74 identified putative pilus biosynthesis genes, which we annotated as *spaCB-spaA-srtC1-srtC2*.

### Plasmid pNZ712 specifies nisin immunity and copper resistance

Nisin is a lanthionine-containing antimicrobial peptide that binds to lipid II, disrupts the cytoplasmic membrane and causes death of susceptible bacterial cells [71]. The nisin operon *nisABTCIPRKFEG* carries, next to the nisin structural gene *nisA*, genes responsible for nisin modification, transport and precursor cleavage, genes involved in nisin operon regulation and genes specifying resistance to the bacteriocin [47,72]. Plasmid pNZ712 carries *nisCIP*, albeit *nisC* is only partially present. The *nisC* gene product NisC, in concerted action with NisB, is involved in posttranslational modification of the nisin precursor. The *nisI* gene specifies nisin immunity, while *nisP* encodes the serine protease involved in maturation of the nisin precursor.

To determine whether pNZ712 *nisI* is functional, *L*. *lactis* NCDO712 was grown in LM17 medium supplemented with 0 or 20 ng/ml nisin. *L*. *lactis* NZ9700 was used as a nisin resistant control. All strains reached a maximal OD_600_ of 2.96±0.63 after 7 h of growth without nisin. When they were grown in the presence of 20 ng/ml nisin, the positive control strain NZ9700 reached an OD_600_ of 1.8±0.06. *L*. *lactis* NCDO712 and SH4109, two strains carrying all 6 plasmids including pNZ712 (*nisCIP*), reached an OD_600_ of 0.63±0.01 and 0.67±0.09, respectively. The latter strains reached lower final optical densities than strain NZ9700, which has full immunity function as it carries *nisFEG* genes encoding an ABC transporter [73] that contributes to nisin immunity *nisI* in NCDO712. The plasmid-free *L*. *lactis* MG1363 and other derivatives carrying single plasmids from *L*. *lactis* NCDO712, but not pNZ712, reached an OD_600_ of only 0.06±0.046. From these results we conclude that the pNZ712-encoded *nisI* is functional and provides nisin resistance. The production of nisin has been suggested to give an advantage to *L*. *lactis* in a plant environment where *nis* genes often co-occur with sucrose utilization genes [74]. The nisin operon seems less prevalent in dairy isolates, indicating a limited benefit in this environment. The constitution of the starter culture from which *L*. *lactis* NCDO712 was isolated is unknown, but if this culture would have contained a nisin producer the maintenance of the *nisCIP* genes could have given an advantage.

Plasmid pNZ712 also carries a set of genes, *lcoRSABC*, which may be involved in copper resistance. Plasmid-encoded copper resistance has been described previously in *Streptococcus* (now *Lactococcus*) *lactis* [75,76], but has not been described for *L*. *lactis* NCDO712. The genes *lcoRS* are involved in transcription regulation of *lcoABC*, the products of which confer copper resistance by lowering the accumulation of copper inside the lactococcal cell [7]. Furthermore, pNZ712_*23* encodes a putative copper-(or silver)-translocating P-type ATPase.

To examine whether pNZ712 specifies copper resistance, *L*. *lactis* NCDO712 and several of its derivatives were grown with or without CuSO_4_. *L*. *lactis* MG1363 and other NCDO712 derivatives did not grow when 0.8 mM or more CuSO_4_ was present in the medium. *L*. *lactis* NCDO712 was the only of the tested strains that was able to grow in the presence of up to 4 mM (1 g/L) CuSO_4_, indicating that the *lcoRSABC* gene cluster and/or pNZ712*_23* on pNZ712 are functional. The minimum inhibitory concentration of CuSO_4_ was1 g/l (S2 Fig). We also showed that pNZ712 harboring the copper- and nisin-resistance genes can be transferred from NCDO712 to MG1614 via conjugation using copper as a selective marker. The conjugation frequencies were 8.1e-8 ± 2.6e-8 transconjugants per donor cell and using PCR we confirmed that only pNZ712 was transferred to the recipient strain.

Interestingly, a recently diverged derivative of *L*. *lactis* NCDO712, strain C2 [69,77], was reported to carry 5 plasmids. The resistance of this strain to copper and other metal ions was suggested to be encoded by the lactose plasmid pLM3001 (approximately 30 MDa), of which the nucleotide sequence has not been determined [75]. Our results, showing that copper resistance and lactose utilization genes reside on different plasmids in strain NCDO712, indicate that plasmid re-arrangements may have occurred in either or both of these strains [77].

### A novel pilus gene cluster is present on pSH74

**Fig 2:**
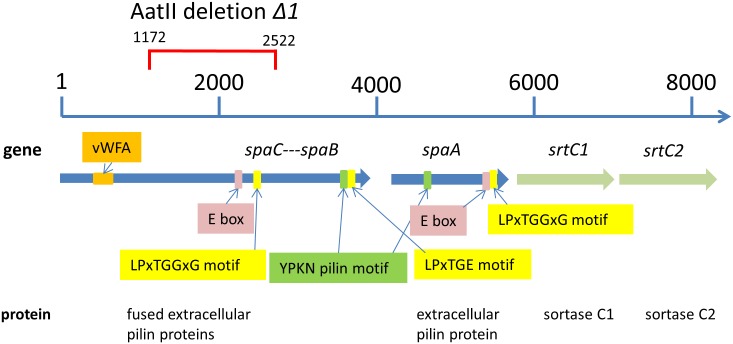
Schematic representation of the pilus gene cluster on pSH74 and the suggested functions of its constituting genes (not to scale). The internal deletion of 1451 bases in *spaCB* is indicated in red.

Plasmid pSH74 harbors an 8-kb gene cluster, which we annotated as *spaCB-spaA-srtC1-srtC2* (Fig 2). The order of genes in pilus biosynthesis clusters differs among gram-positive bacteria [39,78]. The synteny on pSH74 resembles that of the *spaC-spaB-spaA-srtC1* cluster of *Lactobacillus rhamnosus* GG most [42,43,79]. However, *spaC* and *spaB* seem to be fused in *L*. *lactis* NCDO712 (see below) and the operon-encoded proteins display only 30 to 45% amino acid sequence identity. An additional difference to *L*. *rhamnosus* GG is that the GG pilus cluster contains only one sortase gene while the pSH74 pilin gene cluster contains two adjacent *srtC* genes encoding SrtC enzymes of 413 and 392 amino acid residues, respectively. These show only 38% mutual amino acid identity. Two or even three consecutive *srtC* genes have previously been identified in the pili biosynthesis gene clusters in *Streptococcus agalactiae*, *S*. *pneumoniae*, and *Clostridium diphteriae* [80,81].

The GC content in the NCDO712 *pil* operon (35.3%) closely matches that of the NCDO712 chromosome (35.7%) suggesting that its acquisition is not a recent event or that it has been transferred from a microbial species with a similar GC content. The latter is consistent with the presence of a highly homologous pilus gene cluster in the genome of *Leuconostoc citreum* (> 90% protein sequence identity) with a GC content of 38.9%.

Previously two other pilus gene clusters were identified in *L*. *lactis*. Oxaran and co-workers [22] identified a chromosomally encoded pilus gene cluster in *L*. *lactis* IL1403, *yhgD*, *yhgE*, *yhhA*, *yhhB*. This chromosomal cluster, which does not show homology at the protein or synteny level to the pSH74 pilus gene cluster, appears to be present in all lactococcal genomes known to date, including those of *L*. *lactis* strains SK11, KF147, and MG1363 (llmg_1800–1803) [22] as well as NCDO712. Similarly, a plasmid-located 6.9-kb pilus gene cluster *yhgE2*-*srtC2*-*yhhB2*-*ORF4* detected in *L*. *lactis* TIL448 [21] does not show significant homology or synteny to the pilus gene cluster identified here. A sequence comparison between the chromosomal pilus gene cluster in NCDO712 and the plasmid pSH74-located gene cluster identified here showed no synteny. Protein sequence analysis showed that SrtC1 and SrtC2 have a 35% (over 66% of the query) and 32% (over 73% of the query) identity with the chromosomally encoded SrtC (llmg_1801) respectively. Typically less than 30% amino acid identity is observed for the other proteins if the query sequence is at least 20% of the length of the protein. The presence of two pilus gene clusters in one strain suggests that they might have different functions and it raises the question whether *L*. *lactis* NCDO712 is able to produce the two types of pili simultaneously. Previous work has shown that *L*. *pneumophila* AA100 expressed both, long and short pili, but not at the same time [82] while concurrent expression of two types of pili has been reported for *E*. *faecium* [83]. *L*. *lactis* MG1363, which contains the chromosomal *pil* operon, only produces pili on its cell surface when it overexpresses the *pil* operon from pSH74, as is clear from the electron microscopy data and phenotypic characterization presented here. Whether conditions exist in which both types of pili are expressed simultaneously in *L*. *lactis* NCDO712 remains to be determined.

We hypothesize that the functions of the pilin gene products of *L*. *lactis* NCDO712 pSH74 are similar to those in *Lb*. *rhamnosus GG* [80] and other gram-positive bacteria [36,38,84–87]. Based on this hypothesis the SpaA protein of *L*. *lactis* NCDO712, SpaA^Ll^, is presumably the major pilus backbone subunit (Fig 2). It contains the typical C-terminal LPSTGGAG motif involved in sortase C-catalyzed transpeptidation and has the characteristic YPKN “pilin motif”. The YPKN motif is involved in intermolecular peptide bond formation between the carbonyl-group carbon of the threonine residue of the pilin subunit and the side-chain of the lysine in the pilin motif of the neighboring pilin subunit [41]. These bonds lead to the formation of membrane-associated covalently-linked dimers with a pilin motif that can interact with other pilin subunits, forming an elongated pilus fibre [41]. SpaA^Ll^ also carries the YVLNETKAP “E box”, which has a structural role in pilus assembly (52). This was illustrated by the involvement of the “E box” of SpaA from *Corynebacterium diphtheria* in the attachment of SpaB to polymerized SpaA fibres [38].

*Lb*. *rhamnosus* GG SpaB (SpaB^GG^) is the basal pilin subunit and SpaC^GG^ is the pilin tip protein. While *spaC* and *spaB* are separate genes in *Lb*. *rhamnosus* GG, they are fused in *L*. *lactis* NCDO712. The first 840 amino acid residues of *L*. *lactis* NCDO712 SpaCB (SpaCB^Ll^) correspond to SpaC^Ll^, while the remaining 260 residues resemble SpaB^Ll^. The SpaC segment in SpaCB^Ll^ contains an LPSTGGSG motif which is a potential cleavage site for SrtC between the threonine (T) and glycine (G) residue [41], thereby splitting SpaCB^Ll^ into the respective proteins, SpaC^Ll^ and SpaB^Ll^. The SpaB^Ll^ resembling part of SpaCB^Ll^ contains a C-terminal LPDTGE motif (Fig 2B) that is predicted to be targeted by SrtA and serve as a peptidoglycan anchoring sequence [79,80]. Hence, SpaB^Ll^ is most likely at the base of the lactococcal pilus.

The SpaC^Ll^ segment of SpaCB^Ll^ has an “E box” (YALTETKTP), which has a structural role in pilus assembly (14, 61, 78). Possibly, after cleavage of SpaCB^Ll^ by SrtC, the “E box” is used to link SpaC^Ll^ to SpaA^Ll^. The SpaC^Ll^ segment contains a von Willebrand type-A domain (vWFA). It has been speculated that a vWFA domain, which is also present in SpaC of *L*. *rhamnosus* GG [43], may have lectin-like binding properties and could bind to sugars with high carbohydrate specificity. SpaC^Ll^ also carries a collagen-binding domain and two collagen-binding surface-protein-Cna B-type domains, which might be involved in bacterial adhesion to surfaces (IPR008970; SSF49478) (http://supfam.org). Taken together, these observations suggest that, analogous to SpaC^GG^ in the *Lb*. *rhamnosus* GG pilus, SpaC^Ll^ might fulfill the tip protein function.

Based on protein homology searches we identified similar pilus gene clusters in *L*. *lactis* subsp. *cremoris* CNCM I-1631 contig_071 (accession number: AGHX01000000; LLCRE1631_01806, LLCRE1631_01807, LLCRE1631_01808, LLCRE1631_01809) (97% identity), in *L*. *lactis* subsp. *lactis* 1AA59 contig_056 (accession number: AZQT01000047.1 and AZQT01000000; KHE76387.1, KHE76388.1, KHE76389.1, KHE76390.1) (100% identity), as well as in the *Leuconostoc citreum* genome (accession numbers: WP_048699698, WP_048699696, WP_048699695, WP_048699693) (>90% identity). In all these cases, the corresponding protein sequences are annotated as encoding hypothetical proteins. The *spaCB* genes are fused in these strains as well as in *L*. *lactis* NCDO712, suggesting that this might be of relevance for e.g., controlling the ratio of the two proteins in the pilus structure.

Pilus formation and attachment to peptidoglycan in *Lb*. *rhamnosus GG* is governed by the pilin-specific sortase SrtC1 and the house-keeping sortase SrtA. SrtC1 specifically targets a triple glycine motif LPxTGGxG at the N-terminal end of pilin proteins, and catalyzes their assembly into pili [84]. The chromosomally-encoded enzyme SrtA targets N-terminal LPxTGE or LPxTGD motifs [80] and covalently anchors extracellular proteins, including pilin proteins, to peptidoglycan in the cell wall. The SrtA recognition sequence does not necessarily prevent its recognition by a sortase other than SrtA [80]. Thus, SrtA and SrtC1 may each be able to recognize both LPxTGD/E and LPxTGGxG motifs used for pilin polymerization and anchoring [80]. However, the protein-structural features involved in motif recognition by sortases are currently unknown. When *spaA* and *srtC1* of *Lb*. *rhamnosus GG* were co-expressed in *L*. *lactis* NZ9000, it was observed that SrtC1 recognized and polymerized the SpaA protein. *Lb*. *rhamnosus GG* SpaC has the same LPxTG motif as SpaA [80], therefore, SrtC1 possibly recognizes the pilus tip protein SpaC similar to SpaA, while SrtA only anchored SpaB to the peptidoglycan. Based on the similarity of the pSH74-located pilus cluster to that of *Lb*. *rhamnosus* GG, we speculate the gene functions to be similar, namely that one or both of the plasmid encoded C sortases in *L*. *lactis* NCDO712 are responsible for the assembly of pilin proteins in this strain, while the chromosomally encoded SrtA (llmg_1449) covalently anchors the pili to the peptidoglycan of the cell wall [79,80]. Data obtained from RNAseq performed on total RNA isolated from *L*. *lactis* NCDO712 growing under different stress conditions revealed that *srtC1* and *srtC2* are usually co-expressed, while there is no correlation in expression levels between the other genes of this gene cluster [67].

### The pilus gene cluster on *L*. *lactis* NCDO712 pSH74 is functional

**Fig 3:**
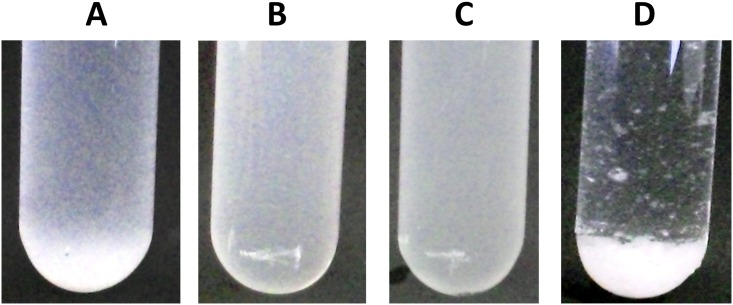
Cell aggregation after pilus gene over-expression. A—*L*. *lactis* NCDO712, B—empty vector control MG1363(pIL253), C—MG1363(pIL253*pilΔ1*), D—MG1363(pIL253*pil*). The images were taken 3 min after re-suspension of cells from an overnight culture.

**Fig 4:**
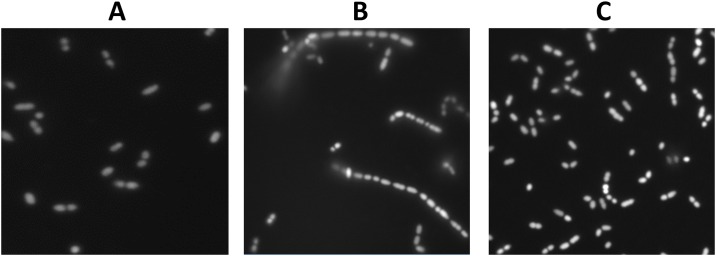
Pilin gene overexpression in *L*. *lactis* MG1363 leads to increased chain length. A—MG1363(pIL253), B—MG1363(pIL253*pil*), C—MG1363(pIL253*pil*Δ*1*).

To examine whether the identified putative pilin genes are functional, the entire pilus gene cluster with its native promoter was cloned in the medium-copy number (45–85) plasmid pIL253 [52], resulting in pIL253*pil*. Initial attempts to clone *pil* downstream of the nisin-inducible *nisA* promoter or the constitutive *purC* promoter failed. The few clones that were obtained all carried the same internal deletion in the *spaCB* genes. Deliberate deletion of a similar internal 1.5 kb fragment resulted in pIL253*pil*Δ*1* (Table 1, Fig 2). The deletion lead to a frame-shift mutation shortening the SpaCB^Ll^ protein by 744 amino acid residues and resulting in a 394-residue truncated SpaC protein. This protein still carries the vWFA domain but the YPKN pilin motif, the LPDTGE motif (Fig 2), the “E box” and LPS amino acids of LPSTGGSG motif were deleted. RNAseq data revealed that the native promoter includes a leader sequence of ~200 nt upstream of *spaCB* [67]. Although the presence of this leader seems important for the successful over-expression, its precise role is currently not known. Both plasmids pIL253*pil* and pIL253*pil*Δ*1* were introduced in *L*. *lactis* strains MG1363 and IL1403. The observed pilus over-expression from the pIL253-based vector resulted in cell aggregation and rapid sedimentation of the cultures of both strains (Fig 3). The cells also grew in chains containing more than 10 cells, while the wild type strain MG1363 and control strain MG1363pIL253 did not form such chains (Fig 4). Cells expressing the truncated version of SpaCB^Ll^ displayed neither cell aggregation (Fig 3) nor cell chaining (Fig 4). As the anchoring of pili to the cell surface is expected to be essential for their functionality the effect of the *spaCB* truncation is consistent with the loss of cell aggregation and chaining.

**Fig 5:**
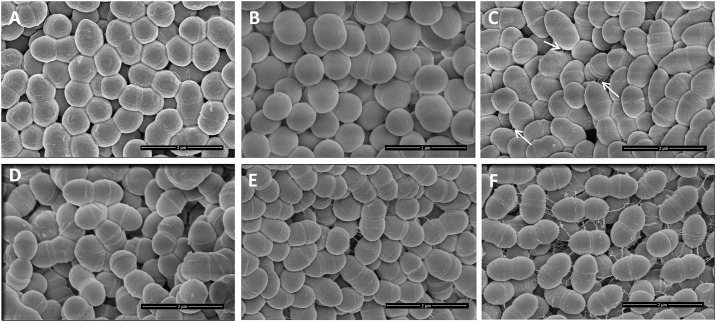
Scanning electron microscopy at 50.000 x magnification of *L*. *lactis* strains over-expressing *spaCB*-*spaA*-*srtC1*-*srtC2*. A—NCDO712, B—IL1403, C—IL1403(pIL253*pil*), D—MG1363(pIL253), E—MG1363(pIL253*pil*), F—MG1363(pIL253*pil*Δ*1*). White arrows indicate pili in panel C. Black bars: 2 μm in all panels.

**Fig 6:**
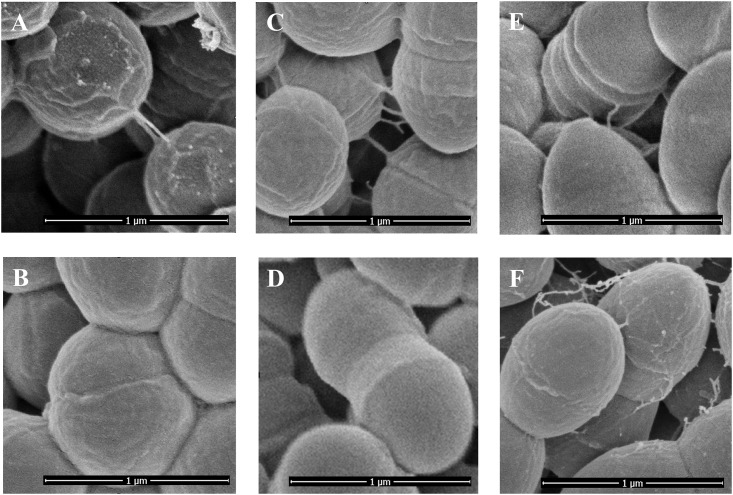
Scanning electron microscopy of *spaCB*-*spaA*-*srtC1*-*srtC2* over-expressing *L*. *lactis* strains. A—NCDO712(pIL253*pil*), B—NCDO712, C—MG1363(pIL253*pil*), D—MG1363(pIL253), E—IL1403(pIL253*pil*), F- MG1363(pIL253*pilΔ1)*. Black bars: 1 μm in all panels.

Scanning electron microscopy (SEM) revealed that *L*. *lactis* NCDO712 (Fig 5A) cells have a relatively rough surface while those of *L*. *lactis* IL1403 were very smooth (compare A and B in Fig 5). The introduction of pIL253*pil* resulted in an increased roughness of the cell surface for both for IL1403 and MG1363. Furthermore, even though *L*. *lactis* NCDO712 harbors the *pil* operon on pSH74, pili were not visible on the surface of these bacteria. This indicates that expression of pili from the native plasmid, estimated to be present at 3 copies per cell (Table 2), is not high enough to allow their detection. However, expression of the same operon (with the native promoter) from plasmid pIL253, which is present in higher (45–85) copy numbers [52], is sufficient to detect pilus-like structures on cells of *L*. *lactis* IL1403(pIL253*pil*) (Figs 5C and 6E), MG1363(pIL253*pil*) (Figs 5E and 6C) and NCDO712(pIL253*pil*) (Fig 6A). Similar to what we found in NCDO712, detection of pili from the *L*. *lactis* IL1403 chromosomal pilus gene cluster *yhgD*, *yhgE*, *yhhA*, *yhhB* could only be achieved by their overexpression [22]. However, in *L*. *lactis* isolates from vegetables, such as KF282 and NCDO2118, and in the clinical isolates 2885–86 and 810–85, pili were detectable under standard growth conditions using negative staining and TEM analysis [22]. In another study wild-type *L*. *lactis* TIL448 was shown to carry a plasmid-encoded 6.9-kb pilus gene cluster, *yhgE2-srtC2-yhhB2-ORF4*, that lead to the formation of short (260–440 nm) and low density pili at the cell surface detectable with AFM [21]. Overexpression of the *L*. *lactis* TIL448 pilus gene cluster *yhgE2*-*srtC2*-*yhhB2*-*ORF4* in *L lactis* MG1363 (TIL1293) led to thicker (2.3 nm), longer (1.2–2.5 μm) and higher density pili at the cell surface [21].

Interestingly, MG1363(pIL253*pil*Δ*1*) cells expressing the truncated version of SpaCB^Ll^ (Figs 2 and 5F) were also decorated with pilus-like structures, but these appeared to be more disoriented (Fig 5, compare E and F), which would be consistent with SpaA being the shaft pilin. This disorientation may imply that the pili produced by MG1363(pIL253*pil*Δ*1*) are not appropriately attached to the peptidoglycan, which is consistent with the truncated form of SpaCB^Ll^ lacking the predicted SrtA motif for peptidoglycan anchoring. All in all, the results reveal that the *spaCB*-*spaA*-*srtC1*-*srtC2* pilus gene cluster from pSH74 of *L*. *lactis* NCDO712 is functional and leads to the formation of pilus-like appendices on the surface of lactococcal cells.

The *Lactobacillus rhamnosus* GG pilus operon *spaC-spaB-spaA-srtC1* is involved in attachment to human epithelial colorectal adenocarcinoma cells [43], and an intestinal mucus-binding capacity was attributed to the tip pilin subunit SpaC [42] via mucus-binding domain. *Lb*. *rhamnosus GG* also carries a pilus gene cluster, *spaFED*, on its chromosome, where SpaD is the pilus backbone protein, SpaE is at the base of the pilus structure, and SpaF is the minor pilin subunit that locates to the tip of the pilus. SpaF was also shown to be responsible for adhesion of pili-carrying cells to the intestinal mucus [42,88]. Furthermore, cloning of *spaC*^*GG*^, *spaB*^*GG*^, *spaD*^*GG*^, *spaE*^*GG*^, *spaF*^*GG*^ in *Escherichia coli* and assessment of the adherence of these proteins to human intestinal mucus reveled that also the SpaB pilin subunit plays a role in binding to intestinal mucus, through electrostatic contacts [42]. In the *L*. *lactis* TIL448 pilus gene cluster *yhgE2*-*srtC2*-*yhhB2*-*ORF4*, the latter is the putative pilus tip protein. It contains a lectin-like domain (PF00139) predicted to have carbohydrate-binding properties, which could be involved in binding to plant cell walls [21]. Furthermore, over-expression of TIL448 *yhgE2*-*srtC2*-*yhhB2*-*ORF4* in *L*. *lactis* MG1363 (TIL1293) was shown to increase attachment of the bacteria to Caco-2 cells [21]. No significant differences in attachment to Caco-2 and HT29 (a human colonic carcinoma cell line) cells were observed when stationary-phase cells of *L*. *lactis* with or without pIL253*pil* were tested (S5 Table). Similar to our results earlier studies also described that strain NCDO712 (synonym C2 or TIL256) did not adhere to Caco-2 cells [21].

In gram-negative bacteria the length of pili is approximately 1–2 μm, and the diameter is between 1 nm and 10 nm [36,41]. In gram-positive bacteria pili are more varied with reported lengths of 0.3–3 μm, and a diameter between 3–10 nm [41]. The *Lb*. *rhamnosus* GG *spaC-spaB-spaA-srtC1* pili are up to 1 μm in length, with a diameter of 1 to10 nm [43,79]. The pili encoded by *yhgD*, *yhgE*, *yhhA*, *yhhB* in *L*. *lactis* IL1403 are up to 3 μm long and have a diameter of approximately 5 nm [22]. Pili on the surface of wild type *L*. *lactis* TIL448 specified by *yhgE2*-*srtC2*-*yhhB2*-*ORF4* were short (up to 450 nm) and thin, but the pili of the overexpressing *yhgE2*-*srtC2*-*yhhB2*-*ORF4* cluster strain *L*. *lactis* MG1363 (TIL1293) were longer with an average length of 2 μm and diameter of approximately 2.3 nm [21]. Interestingly, the pili overexpressed by MG1363(pIL253*pil*) are shorter and thicker than earlier reported lactococcal pili [21,22,24], having a length of 200–240 nm and a diameter between 18 and 20 nm. The appendages produced by MG1363(pIL253*pil*Δ*1*) were thinner and had an average diameter of approximately 14 nm and the length of 120–560 nm.

### Conjugation efficiency

One of the roles of certain pili in gram-negative bacteria is to enable conjugation [89]. The overexpression of the pili identified here frequently displayed cell-cell contact (Fig 6A and 6C), which led us to examine whether the *L*. *lactis* NCDO712 *pil* operon might play a role in DNA transfer by conjugation. *L*. *lactis* NCDO712 and MG1363 carry a 50-kb sex factor in its chromosome that is involved in co-integrate formation with and subsequent conjugal transfer of the lactose/protease plasmid pLP712 [29,35]. This process can be readily quantified using the plasmid-free, lactose-deficient *L*. *lactis* MG1614 (Lac^-^, PrtP^*-*^, Strep^R^, Rif^R^) (Table 1) as a recipient and selecting for lactose-fermenting colonies that are also resistant to rifampicin and streptomycin. The efficiency of transfer of pLP712 from strains NCDO712 and MG1299 was up to 22 and 16 fold increased respectively, when the strains carried pIL253*pil* and, thus overexpressed pili (S6 Table). We did not observe co-transformation of pIL253*pil* during the conjugation of pLP712. Although the observed increase in the frequency of conjugation is limited, the observations indicate that the pili contribute to the efficacy of exchange of DNA between lactococcal cells. Whether this effect is caused by pili-mediated cell clumping and, with that, increased cell-cell contact (Fig 6), or whether the pili are involved in actual DNA transfer requires further investigation.

## Conclusion

Total DNA sequencing of *L*. *lactis* NCDO712 revealed functional, plasmid-encoded nisin immunity and copper resistance genes as well as a novel pilus gene cluster (*spaCB-spaA-srtC1-srtC2*). Based on bioinformatic analyses and experimental results we predict the pilin tip protein to be SpaC, the basal subunit to be formed by SpaB and SpaA to represent the backbone protein. While surface decoration of bacterial cells with pilus-like structures is widespread and functions such as DNA transfer [37], surface attachment and biofilm formation [22] or interaction with host cells[21] have been described [36], it is still unclear what the function(s) of pili in lactococci is. Pilus expression occurs under standard laboratory conditions in lactococcal strains isolated from plant material or from humans [22], but this is not the case for any of the investigated dairy isolates [1,7,25]. Pili of plant-associated *Pseudomonas syringae* are involved in the attachment of the bacteria to the plant surface [44]. It remains to be determined whether pili in dairy lactococci are evolutionary remnants from their proposed plant ancestry [90] or whether they fulfill a function in the dairy environment.

## Supporting Information

S1 Fig Plasmid identity to pNZ712. Five out of 100 plasmids with high partial sequence identity to pNZ712 are shown. The similar regions include genes encoding copper resistance genes *lcoRSAB*, mobilization genes *mobD*, *mobC* and a gene involved in replication (*repA)* with 99% (yellow) and 93% (grey) identity (see also S4 Table). *L*. *lactis* pND306 of *L*. *lactis* subsp. *lactis* 1252D [91] encoding the copper resistance associated *lcoRSABC* genes were almost identical to a similar locus (6.6 kb) encoded by pNZ712 (only 3 SNPs in the 6.6 kb *lcoRSABC* locus), of which also a part (*lcoRSA*) is present on pSK11P from *L*. *lactis* subsp. *cremoris* SK11 [7].
(TIF)

S2 Fig Growth of *L*. *lactis* NCDO712 and its derivatives carrying one or two plasmids (see Table 1) in medium with or without 0.8 mM (0.2 g/L)–4.8 mM (1.2 g/L) CuSO_4_. *L*. *lactis* NCDO712 is the only strain carrying pNZ712 with *lcoRSABC* coding for copper resistance genes. Each curve represents the average of 3 biological replications. Error bars show standard deviation.
(TIF)

S1 Table Oligonucleotides used in this study. (PDF)

S2 Table Single nucleotide polymorphisms between the chromosomes of *L*. *lactis* NCDO712 and its plasmids-free derivative MG1363 [66]. (PDF)

S3 Table Main plasmid genes and features. (PDF)

S4 Table The main alignment results of pNZ712 and pSH74 to known lactococcal plasmids. (PDF)

S5 Table Microbial adhesion to HT29 and Caco cells given in percent cells recovered after incubation with the cell lines. Standard deviation is given in parenthesis (n = 3).
(PDF)

S6 Table Effect of the pilus gene cluster on conjugation of the lactose-fermenting ability to *L*. *lactis* MG1614. (PDF)
