# Supplementary figures and images for: Plasmid Complement of Lactococcus lactis NCDO712 Reveals a Novel Pilus Gene Cluster
Source: PLoS One. 2016 Dec 12;11(12):e0167970. doi: 10.1371/journal.pone.0167970 (PMC5152845; doi:10.1371/journal.pone.0167970)

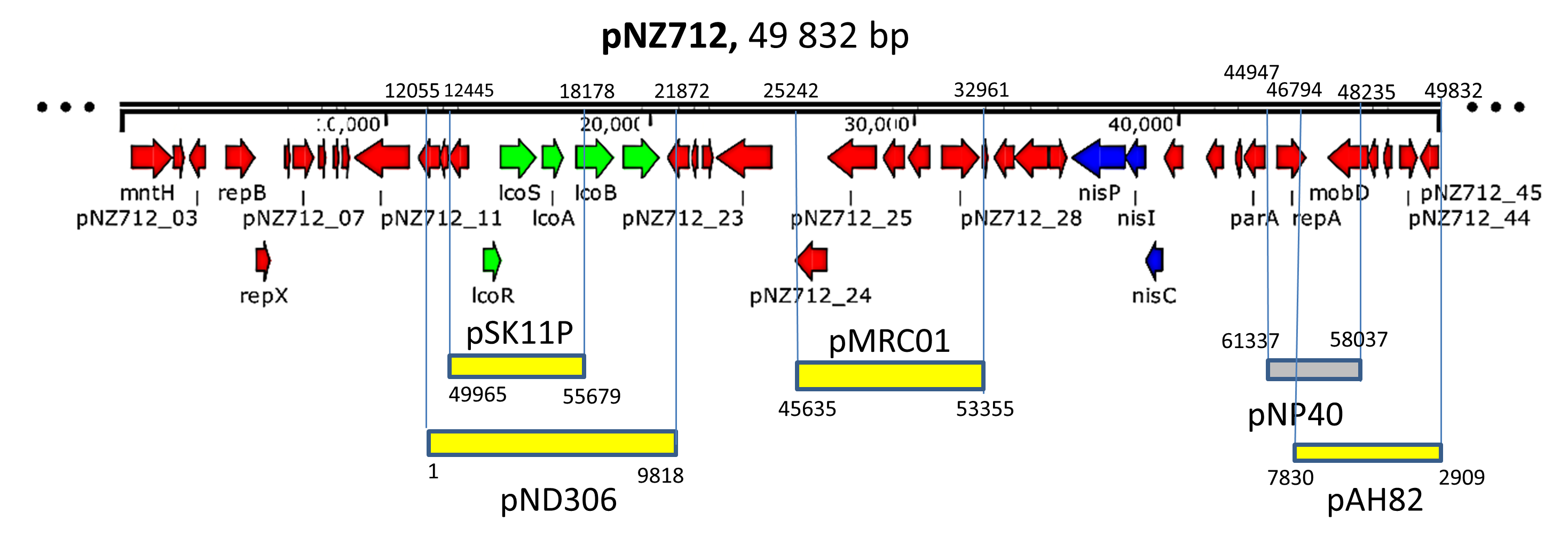

Supplement: S1 Fig — Five out of 100 plasmids with high partial sequence identity to pNZ712 are shown. The similar regions include genes encoding copper resistance genes lcoRSAB, mobilization genes mobD, mobC and a gene involved in replication (repA) with 99% (yellow) and 93% (grey) identity (see also S4 Table). L. lactis pND306 of L. lactis subsp. lactis 1252D [91] encoding the copper resistance associated lcoRSABC genes were almost identical to a similar locus (6.6 kb) encoded by pNZ712 (only 3 SNPs in the 6.6 kb lcoRSABC locus), of which also a part (lcoRSA) is present on pSK11P from L. lactis subsp. cremoris SK11 [7]. (TIF) [file pone.0167970.s001.tif]

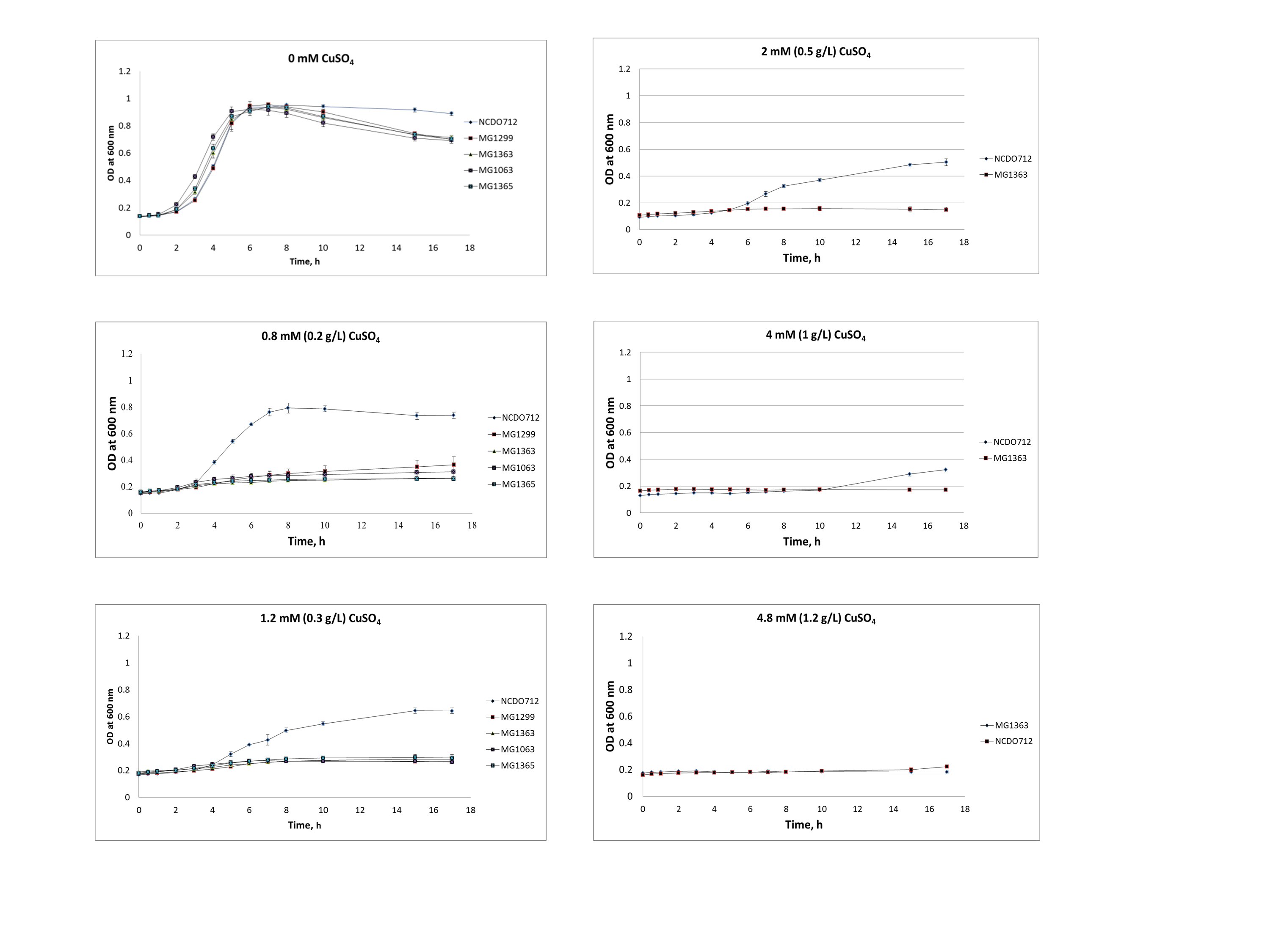

Supplement: S2 Fig — L. lactis NCDO712 is the only strain carrying pNZ712 with lcoRSABC coding for copper resistance genes. Each curve represents the average of 3 biological replications. Error bars show standard deviation. (TIF) [file pone.0167970.s002.tif]
