# Supplementary material for: Plasmid Complement of Lactococcus lactis NCDO712 Reveals a Novel Pilus Gene Cluster
Source: PLoS One. 2016 Dec 12;11(12):e0167970. doi: 10.1371/journal.pone.0167970 (PMC5152845; doi:10.1371/journal.pone.0167970)
Supplement: S1 Table — (PDF) [file pone.0167970.s003.pdf]

S1 Table. Oligonucleotides used in this study

A) To identify *L. lactis* NCDO712 plasmids (34)	
pLP712_FW	GCTTTAATGGCTGCTCCATC
pLP712_RV	AGCACACCCGGATGATAGTC
pSH73_FW	TTTCAGTAGAAGGCCAAACAAC
pSH73_RV	TGCAAATTTATCTACAAAGGCTTG
pSH72_FW	GCTTTTTCGTTGGTTTGCTC
pSH72_RV	GCCCAAAATAGTGGGTTAGTG
pSH71_FW	TTGGGATAGAGCGTTTTTGG
pSH71_RV	CGGGGGAAATAAAATGACAAAC
pSH74_FW	GGACCAGATGGTACTTTTGAAGCG
pLSH74_RV	GGTAAAGTCACTATTGATGGACAGCC
pNZ712_FW	CACTCTAGTTTCCTACCTTCGTTGCAAGC
pNZ712_RV	CCAGTGCTAATCCTCCGTATAAGTATAGC
B) For pilin operon cloning	
pilin*PstI* forw*	CCGctgcagTTTGCAACAGAACCGTAATTGATTAGC
pilin*XhoI* rev*	CGGctcgagTTAAGTAATTTGAATTACTTGCTTTGAGAGTTGT TTAAAGG

*Indicated restriction enzyme sites are shown in lower case letters in the oligonucleotide sequence.
