# Supplementary material for: Plasmid Complement of Lactococcus lactis NCDO712 Reveals a Novel Pilus Gene Cluster
Source: PLoS One. 2016 Dec 12;11(12):e0167970. doi: 10.1371/journal.pone.0167970 (PMC5152845; doi:10.1371/journal.pone.0167970)
Supplement: S2 Table — (PDF) [file pone.0167970.s004.pdf]

S2 Table. Single nucleotide polymorphisms between the chromosomes of *L. lactis* NCDO712 and its plasmids-free derivative MG1363 (72).

Predicted mutations					
**Position$**	**Mutation**	**Annotation***	**Gene**	**Description**	**SNP type#**
26455	T→C	Intergenic (+111/-39)	*llmg_tRNA_07* → / → *mtlA*	tRNA-Asn/PTS system mannitol-specific transporter subunit IIBC	Intergenic, predicted to change promoter at position -39; the -10 box is altered from an optimal TATAAT into TACAAT
522850	G→T	S214S (TCG→TCT)	*glpT* →	glycerol-3-phosphatase transporter	Synonymous
668056	A→C	D155E (GAT→GAG)	*tnp1297* ←	transposase for insertion sequence element IS1297	Non-synonymous; SIFT score - 1
671366	G→T	Q2K (CAA→AAA)	*llmg_0678* ←	transposase helper protein for IS981	Non-synonymous; SIFT score – 1
671428	T→C	intergenic (-59/-71)	*llmg_0678* ← / → *llmg_0679*	transposase helper protein for IS981/hypothetical protein	Intergenic
1167414	T→G	Intergenic (+64/-55)	*llmg_1198* → / → *llmg_1200*	hypothetical protein/hypothetical protein	Intergenic
1272351	C→T	L84L (TTG→TTA)	*llmg_1303* ←	hypothetical protein	Synonymous
1529872	A→C	I36M (ATT→ATG)	*llmg_1557* ←	hypothetical protein	Non-synonymous; SIFT score – 0.79
2143208	T→G	S244S (TCT→TCG)	*tnp904* →	transposase for insertion sequence element IS904I	Synonymous
2380149	C→T	G118D (GGC→GAC)	*tsf* ←	elongation factor Ts	Non-synonymous; predicted to affect protein function; SIFT score – 0.01
2492669	A→C	V284G (GTT→GGT)	*gapB* ←	glyceraldehyde 3-phosphate dehydrogenase	Non-synonymous; predicted to affect protein function; SIFT score - 0.02

$ Indicates nucleotide position in the genome sequence of *L. lactis* MG1363 (RefSeq: NC_009004.1)

* For mutations in intergenic regions the numbers indicates the relative position to the neighboring genes. For mutations in genes the amino acid (top) and nucleotide (bottom) changes are given.

The amino acid substitution is predicted to be deleterious if the SIFT score is <= 0.05, and tolerated if the SIFT score is > 0.05. If intergenic mutations effect either the -10 or -35 box of the promoter it is indicated
