# Supplementary material for: Plasmid Complement of Lactococcus lactis NCDO712 Reveals a Novel Pilus Gene Cluster
Source: PLoS One. 2016 Dec 12;11(12):e0167970. doi: 10.1371/journal.pone.0167970 (PMC5152845; doi:10.1371/journal.pone.0167970)
Supplement: S3 Table — (PDF) [file pone.0167970.s005.pdf]

S3 Table. Main plasmid genes and features.

Plasmid	Genes	Product/Function
pLP712	*prtP*	subtilisin-like serine protease
	prtM	peptidyl-prolyl isomerase
	dld	D-lactate dehydrogenase
	lacR-lacABCDFEGX	Enzymes involved in lactose degradation
	pepF	Oligoendopeptidase F
	parAB	Plasmid partitioning proteins
	rep	Replication protein
pSH71	*repABC*	Replication proteins
pSH72	*repBXC*	Replication proteins
pSH73	*hsdS*	Type I R/M system specificity subunit
	repXB	Replication-associated protein RepX, RepB
	cadC, *cadA*	Resistance to heavy metals (cadmium), cadmium efflux ATPase (CadA)
pSH74	IS*1216*	1 IS element
	spaCB, spaA, srtC1, srtC2	Pilin gene cluster
	repB, repX	Replication proteins
	hsdS	Type I R/M system, specificity subunit
pNZ712	*nisCIP*	Nisin immunity
	lcoRSABC	Copper resistance
	mobD, *mobC*	Relaxase/mobilisation nuclease MobD and mobilization protein MobC
	repB, repX, repA	Replication proteins
	parA	Plasmid partitioning protein ParA
	mntH	Mn2+/Fe2+ transporter, NRAMP family
	five IS*S1*, three IS*981*, three IS*LL6*, two IS*1076*/IS*1069*/IS*1068*/IS*904*, IS*1251*, IS*712*, IS*1216*, IS*6770*	17 IS elements
