# Supplementary material for: Plasmid Complement of Lactococcus lactis NCDO712 Reveals a Novel Pilus Gene Cluster
Source: PLoS One. 2016 Dec 12;11(12):e0167970. doi: 10.1371/journal.pone.0167970 (PMC5152845; doi:10.1371/journal.pone.0167970)
Supplement: S4 Table — (PDF) [file pone.0167970.s006.pdf]

S4 Table. The main alignment results of pNZ712 and pSH74 to known lactococcal plasmids.

NCDO712 plasmid	Position	Alignmet with plasmid	Position	Sequence identity in %/SNPs	Annotation
pNZ712	12054...21871; 1868...22735	pND306 (accession NG_035558.1) of *L. lactis* subsp. *lactis* 1252D (93)	1….9818; 9782….10650	99%, 3 SNPs, 1 gap	Copper resistance associated *lcoRSABC* genes
	12444…18177	pSK11P (accession NC_017500.1 and NC_008505.1) from *L. lactis* subsp. *cremoris* SK11 (8)	49965…55679	99%, 19 gaps and 8 SNP	*lcoRSA* genes
	46793…49826	pAH82 (accession AF243383)	7830…4798	99%, 3 gaps and 17 SNPs	CDS of unknown function
	25241…32960	pMRC01 from *L. lactis* DPC3147	45635…53355	99%, 3 gaps and 20 SNPs	Hypothetical and putative proteins
	44946…48234	pNP40 (accession DQ534432, AY530537)	61337…58037	93%, 50 gaps and 170 SNPs	*mobD*
pSH74	6395...6959	pKP1 (accession NC_016042.1)	8427...8991	99%, 6 SNPs	Transposase IS*1216*
	6398... 6959	pK214 (accession NC_009751.1) from *L. lactis* K214	29824...29263	99%, 6 SNPs	*tnpA*
	6395...6959	pSK11B (accession NC_013551)	9605…9044	99%, 6 SNPs	*tnp1-IS6*
	4793…5487	pCV56A (accession NC_017483.1) from *L. lactis* CV56	8618…9312	99%, 10 SNPs	Acetyltransferase
	6398…6959	pIBB-JZK (accession NC_024965.1) of *L. lactis* ILIBB-JZK	8369…7808	99%, 4 SNPs	Transposase IS*1216*
	5564...6310	pGdh442 (accession NC_009435) of *L. lactis* NCDO1867	33352…32607	98%, 13 SNPs, 3 gaps	Transposases *tnpR*
	5563...6275	pCIS5 (accession NC_019432) from *L. lactis* UC509.9	7678…8390	99%, 8 SNPS	Resolvase/integrase
