# Supplementary material for: Plasmid Complement of Lactococcus lactis NCDO712 Reveals a Novel Pilus Gene Cluster
Source: PLoS One. 2016 Dec 12;11(12):e0167970. doi: 10.1371/journal.pone.0167970 (PMC5152845; doi:10.1371/journal.pone.0167970)
Supplement: S5 Table — Standard deviation is given in parenthesis (n = 3). (PDF) [file pone.0167970.s007.pdf]

Strains	HT29 (% attachment)	Caco-2 (% attachment)
***Lb. rhamnosus* LGG**	2.3±0.7	1.2±0.8
**Lactococcus lactis NCDO712**	0.3±0.2	2.4±2.2
***Lactococcus lactis* NCDO712(pIL253*pil*)**	0.06±0.06	0.04±0.01
**Lactococcus lactis MG1363pIL253**	0.5±0.05	0.8±0.7
**Lactococcus lactis MG1363(pIL253*pil*)**	0.2±0.1	0.3±0.3
**Lactococcus lactis MG1363**	1.8±0.5	2.8±0.2
**Lactococcus lactis MG1299**	0.6±0.1	2.1±0.4
**Lactococcus lactis MG1063**	0.5±0.2	2.5±1
**Lactococcus lactis MG1365**	0.9±0.5	2.5±0.8
