# Supplementary material for: Plasmid Complement of Lactococcus lactis NCDO712 Reveals a Novel Pilus Gene Cluster
Source: PLoS One. 2016 Dec 12;11(12):e0167970. doi: 10.1371/journal.pone.0167970 (PMC5152845; doi:10.1371/journal.pone.0167970)
Supplement: S6 Table — (PDF) [file pone.0167970.s008.pdf]

S 6 Table. Effect of the pilus gene cluster on conjugation of the lactose-fermenting ability to *L. lactis* MG1614.

Experiment #	Strain	Transconjugants per donor		Fold increased conjugation efficiency
		**Wild type strain**	**Strain harbouring pIL253*pil***	
1	NCDO712	1.90E-07	2.50E-06	13.16
2	NCDO712	1.40E-07	6.00E-06	42.86
3	NCDO712	5.00E-08	4.60E-07	9.20
			**Average fold increase**	**21.74**

Experiment #	Strain	Transconjugants per donor		Fold increased conjugation efficiency
		**Wild type strain**	**Strain harbouring pIL253*pil***	
1	MG1299	3.10E-07	1.60E-06	5.16
2	MG1299	1.50E-07	9.30E-07	6.20
3	MG1299	5.30E-08	1.90E-06	35.85
			**Average fold increase**	**15.74**
